# How Do Information Content and Language Style Relate to Disaster Information Dissemination? The Moderating Role of User Involvement

**DOI:** 10.3390/bs16081362

**Published:** 2026-08-09

**Authors:** Jiaqi Chen, Yanxia Lu

**Affiliations:** 1 School of International Business, Dalian Minzu University, Dalian 116600, China; 20241625@dlnu.edu.cn; 2 School of Management, Liaoning Normal University, Dalian 116029, China

**Keywords:** disaster information dissemination, information content, language style, user involvement, elaboration likelihood model, construal level theory, social media

## Abstract

Social media has become an important channel for information exchange during sudden disasters, with implications for both public-sector communication and corporate risk communication. However, limited research has examined how substantive message content and linguistic presentation jointly relate to online dissemination behaviour. This issue is also relevant to corporate risk management, as firms must respond to crises while managing stakeholder perceptions. Drawing on the Elaboration Likelihood Model (ELM) and Construal Level Theory (CLT), this study conceptualizes information richness and relevance as central-route cues, and specificity and interactive language as peripheral linguistic cues. User involvement is introduced as a motivational condition that may change the relative weight assigned to these cues. The empirical analysis uses 51,856 Weibo posts concerning the 2025 Tibet earthquake. OLS results show that all four message characteristics are positively associated with dissemination intensity, and that user involvement strengthens the associations of content-related cues while weakening those of linguistic cues. A supplementary negative binomial regression confirms the positive associations of information richness and interactive language, as well as the key interaction patterns, though the results for information relevance and specificity are less stable. These findings extend crisis communication research by showing that disaster-information dissemination is jointly associated with substantive content, linguistic presentation, and user involvement, and offer practical implications for organizations seeking to design clearer and more adaptive risk communication.

## 1. Introduction

Sudden disasters create extreme and uncertain information environments in which the public seeks updates, requests assistance, expresses concerns, and shares emergency information. Social media can facilitate the rapid circulation of warnings, rescue requests, and protective guidance, but it may also amplify inaccurate information, emotional reactions, and secondary public-opinion risks (Guo et al., 2022). Weibo is one of the main platforms through which users in China obtain disaster-related information and communicate requests for assistance and material resources (Liu et al., 2025). For enterprises, disasters create multidimensional external shocks, including supply-chain disruption, market volatility, employee-safety risks, and stakeholder communication challenges. Corporate messages therefore serve not only to convey facts but also to manage stakeholder expectations and maintain organizational legitimacy. Managers must decide how to balance substantive completeness with accessible and interactive language, and whether this balance should vary with stakeholder involvement. Understanding why some disaster-related posts receive greater public engagement is therefore important for both crisis communication research and enterprise practice.

Previous studies identified a range of factors associated with crisis-information dissemination, including information sources (Shi et al., 2025), message content (Lu & Qiu, 2024), public emotions (Austin et al., 2023), social contexts (Zhao et al., 2023), and user characteristics (L. Xu et al., 2025). Other research has examined information sharing among organizations and departments during crises (Chang & Huang, 2023) or integrated multiple factors to explain dissemination behaviour (Li et al., 2021). This literature has substantially advanced the understanding of who communicates crisis information, what information is provided, and how the public responds. However, less attention has been paid to how substantive message content and linguistic presentation jointly relate to observable dissemination behaviour.

Information content concerns what a message communicates, whereas language style concerns how that information is expressed. Content-related characteristics, such as richness and relevance, indicate the amount, diversity, and situational value of the information provided. Linguistic characteristics, such as specificity and interactivity, shape how concrete, immediate, and conversational a message appears. Language style can reflect psychological states, social relationships, and communicative intentions (S. Fu et al., 2022). Its role has been examined in online shopping (Moradi et al., 2023), online ratings (Ren et al., 2024), word-of-mouth communication (Zhu et al., 2025), livestream marketing (Hutchinson et al., 2024), doctor–patient communication (S. Wang et al., 2025), and disaster communication (C. H. Lee & Yu, 2020). Nevertheless, crisis communication research has rarely examined information content and language style within a single theoretical and empirical framework.

This separation leaves two theoretical gaps. First, it remains unclear whether content-related and linguistic cues are associated with dissemination through comparable or distinct processes. Information-rich and relevant posts may encourage users to assess the usefulness and situational value of a message. By contrast, specific and interactive expressions may attract attention because they make a post more concrete, accessible, or conversational. Examining either content or language style alone may therefore provide an incomplete account of disaster-information dissemination. Second, previous research has not sufficiently explained how user involvement changes the relative importance of these two types of cues. The Elaboration Likelihood Model (ELM) distinguishes between central and peripheral routes of message processing (Petty & Cacioppo, 1986). When recipients are motivated and able to evaluate a message, they are more likely to scrutinize its substantive information. When motivation or ability is limited, they may rely more heavily on accessible peripheral cues. In sudden-disaster contexts, users differ in the extent to which they regard an event and related information as personally significant. These differences may not simply increase or decrease overall engagement; they may alter the relative weight assigned to message content and linguistic presentation.

The present study uses ELM as the primary framework for distinguishing content-related and linguistic cues. Information richness and information relevance are conceptualized as central-route cues because evaluating them requires attention to the substantive value and applicability of a message. Specificity and interactive language are conceptualized as peripheral linguistic cues because users may respond to their vividness, accessibility, and conversational quality without closely scrutinizing the underlying arguments. The study also draws on Construal Level Theory (CLT) to characterize the representational properties of the linguistic cues (Trope & Liberman, 2010). CLT distinguishes abstract and decontextualized representations from concrete and contextualized representations. Specificity language provides temporal, spatial, quantitative, and situational details, whereas interactive language creates communicative immediacy and interpersonal orientation. CLT therefore helps explain why these linguistic features may become salient during rapid message processing. Psychological distance itself is not directly measured in this study, and the analysis does not test whether language style changes users’ perceived psychological distance.

User involvement is introduced as a motivational condition that may change the relative importance of the two cue types. Users expressing higher involvement may have stronger motivation to evaluate whether disaster-related information is sufficiently detailed, useful, and relevant. Content-related cues should therefore become more diagnostic as involvement increases. In contrast, immediately accessible linguistic cues may become relatively less important when users devote greater attention to substantive information. This mechanism is described as cue reweighting: involvement changes the relative weight assigned to message content and language style rather than uniformly increasing responsiveness to all message characteristics.

Empirically, this study analyses 51,856 Weibo posts concerning the 2025 Tibet earthquake. Because the study uses observational social media data, the estimated relationships are interpreted as conditional associations rather than causal effects. The study addresses the following research questions:

RQ1: How are information content and language style associated with disaster-information dissemination behaviour on Weibo?

RQ2: Do content-related and linguistic cues differ in the magnitude and stability of their associations with dissemination behaviour?

RQ3: How does user involvement change the associations of central-route and peripheral linguistic cues with dissemination behaviour?

## 2. Theoretical Foundation and Research Model

### 2.1. ELM

ELM explains how people respond to persuasive messages through central and peripheral routes (Petty & Cacioppo, 1986). Central-route processing involves closer scrutiny of a message’s arguments and substantive information. It is more likely when recipients are motivated and able to evaluate the message. Peripheral-route processing relies more heavily on readily available cues that allow a judgment to be formed without extensive consideration of the message itself.

The two routes are not mutually exclusive. Individuals may attend to both substantive and peripheral cues, but the relative weight assigned to each depends on motivation, ability, and context. When elaboration likelihood is high, information quality and argument strength become more important. When it is low, salient and easily processed cues may play a larger role. For organizational risk communication, substantive cues generally require more verification and coordination, whereas linguistic cues can be adjusted at lower communication cost; their relative value may therefore vary with stakeholder involvement. ELM has been widely used to explain information processing in digital environments. Previous studies have shown that source characteristics, narrative forms, information quality, and emotional appeals may shape risk perception, attitudes, and behavioural intentions through central and peripheral routes (Y. Xu et al., 2023; S. Zhang et al., 2024; Ouyang et al., 2025). Sudden disasters provide a particularly relevant setting because users face large volumes of rapidly changing information but differ in how carefully they evaluate each post.

In this study, ELM provides the primary framework for distinguishing content-related and linguistic cues. Information richness and relevance are treated as central-route cues because they concern the substantive value and situational usefulness of a message. Specificity and interactive language are treated as peripheral linguistic cues because users may respond to their concreteness, accessibility, or conversational quality without closely evaluating the underlying information. 

### 2.2. CLT

CLT explains how events are mentally represented at different levels of abstraction (Trope & Liberman, 2010). High-level construals are abstract, general, and decontextualized, whereas low-level construals are concrete, detailed, and closely tied to context. These differences in representation may shape how information is understood and evaluated.

CLT has been applied to crisis communication, public behaviour, and digital consumption (Sharples et al., 2023; Kim Saxton et al., 2024; Wu et al., 2023). This literature suggests that the form in which information is presented matters because concrete and contextualized expressions may be easier to visualize and process than abstract descriptions. In disaster communication, specificity language provides temporal, spatial, quantitative, and situational detail. Interactive language creates a sense of interpersonal orientation through collective pronouns, direct references, and interrogative expressions. These features may make a post appear more immediate and accessible during rapid information processing. For organizational risk communication, stakeholder involvement may condition the effectiveness of these linguistic cues.

In the present study, CLT is used only to characterize the representational properties of these linguistic cues. Psychological distance is not directly measured, and the study does not test whether specificity or interactive language changes users’ perceived distance from the event.

### 2.3. Research Model

ELM and CLT perform different but complementary functions in the proposed model. ELM explains how users may respond to substantive and peripheral cues, whereas CLT helps describe the concrete, contextual, and interpersonal properties of the linguistic cues. CLT therefore supplements the interpretation of language style without altering the central–peripheral structure of ELM.

Information richness reflects the amount and diversity of information contained in a post, including textual detail, hyperlinks, and hashtags. Information relevance reflects the extent to which a post addresses the focal disaster and related situational concerns. Because both characteristics require users to consider the value and applicability of the message, they are classified as central-route cues. Specificity language refers to temporal, spatial, numerical, and relational detail, while interactive language refers to expressions that create a sense of participation or exchange. These features shape how a message is presented and can be processed quickly. They are therefore classified as peripheral linguistic cues in the present framework. This classification does not imply that linguistic cues are unimportant or that they cannot be processed carefully. It identifies their primary role in the model: content-related cues provide material for substantive evaluation, whereas linguistic cues shape the accessibility and presentation of that material.

User involvement is introduced as a motivational condition that may change the relative importance of the two cue types. When involvement is high, users may be more willing to assess whether information is sufficiently detailed and relevant to the situation. Under these conditions, content-related cues should become more diagnostic. When involvement is lower, readily accessible linguistic cues may play a larger role in dissemination decisions.

**Figure 1:**
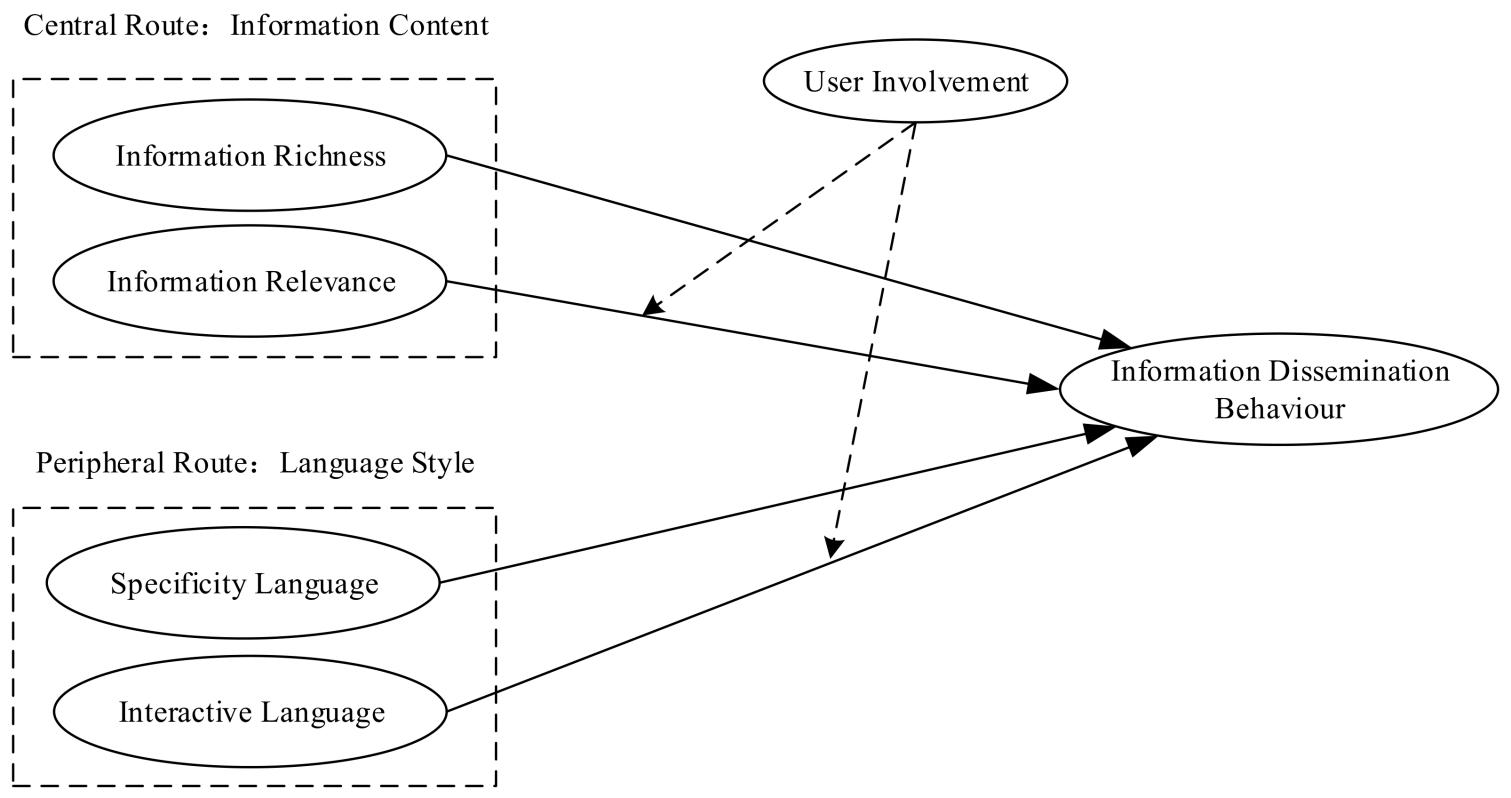
Research model. (Solid arrows represent hypothesized direct effects; dashed arrows represent hypothesized moderating effects).

The proposed mechanism is described as cue reweighting. It does not assume that users rely exclusively on one route or the other. Instead, it suggests that involvement changes the relative weight assigned to content and language style. The empirical model therefore includes four focal message characteristics—information richness, information relevance, specificity language, and interactive language—and tests whether user involvement moderates their associations with disaster-information dissemination behaviour. User characteristics, account characteristics, temporal factors, and IP location are included as controls. The proposed conceptual framework is illustrated in Figure 1.

## 3. Research Hypotheses

### 3.1. Information Content and Information Dissemination Behaviour

Within ELM, central-route processing involves closer evaluation of a message’s arguments and substantive information (Petty & Cacioppo, 1986). This route is especially relevant in sudden disasters, where users must assess rapidly changing information about event development, emergency resources, potential risks, and protective action. Posts that provide sufficient and useful information may therefore offer a stronger basis for deciding whether the message should be reposted or discussed.

Information richness refers to the amount and diversity of information contained in a post. Richer posts may include more textual detail, hyperlinks, hashtags, and contextual information. These elements can help users understand what has happened, where additional information can be found, and whether the message may be useful to others (Y. Wang et al., 2024). In uncertain disaster settings, such information may also reduce ambiguity by offering explanations, evidence, or practical guidance. Richness does not guarantee accuracy or quality. Nevertheless, a post containing more factual and contextual material gives users more information on which to base a dissemination decision. Accordingly,

**H1:**  *Information richness is positively associated with information dissemination behaviour.*

Information relevance concerns the extent to which a post addresses the focal disaster and related situational needs. Relevant information may include event updates, rescue requests, emergency guidance, resource availability, and anticipated developments. Such content is more likely to attract attention because it corresponds to users’ immediate concerns (Mo et al., 2023). From an ELM perspective, relevance increases the diagnostic value of a message. When users perceive that a post addresses the event and its practical consequences, they have a clearer reason to evaluate and redistribute it. Previous crisis communication research has likewise linked message relevance and informational value to public attention and sharing behaviour (L. Zhang et al., 2014). Thus,

**H2:**  *Information relevance is positively associated with information dissemination behaviour.*

### 3.2. Language Style and Information Dissemination Behaviour

Peripheral-route processing relies more heavily on salient and readily accessible cues that allow users to form judgments without extensive scrutiny of the message content (Petty & Cacioppo, 1986). This form of processing is common in fast-moving social media environments, where users often encounter large volumes of disaster-related posts in a short period.

Language style matters in this setting because the same information can be expressed in ways that differ in concreteness, immediacy, and conversational tone. Previous studies have shown that linguistic presentation is associated with public engagement in crisis communication and social media contexts (Sutton et al., 2014; C. H. Lee & Yu, 2020). In this study, specificity and interactive language are treated as peripheral linguistic cues, while CLT is used to describe their representational properties.

Specificity language provides temporal, spatial, quantitative, and contextual detail. A post that states when an event occurred, where it happened, how many people or resources are involved, or what actions should be taken may be easier to understand and visualize than a general statement. Detailed and concrete wording can improve accessibility and persuasive effectiveness (Xie et al., 2024). Specificity may be especially useful during disasters because users often seek information that can be processed and acted on quickly. Posts containing precise locations, times, quantities, or instructions may therefore appear more informative and immediately usable. Accordingly,

**H3:**  *Specificity language is positively associated with information dissemination behaviour.*

Interactive language creates a sense of participation or interpersonal exchange. Collective pronouns, direct references, and interrogative expressions can make a post appear more conversational and may invite users to respond, comment, or share. Research on organizational communication has shown that interactive features are associated with higher levels of online participation (Abitbol & Lee, 2017). In disaster settings, collective and interrogative expressions may similarly draw users into the communicative situation and increase the social salience of a post. Therefore,

**H4:**  *Interactive language is positively associated with information dissemination behaviour.*

### 3.3. The Moderating Role of User Involvement

User involvement refers to the degree to which disaster-related information is personally significant to the user. In ELM, involvement is a central determinant of elaboration likelihood because personally relevant information provides greater motivation for recipients to evaluate a message carefully (Petty & Cacioppo, 1986). Previous studies have found that involvement can strengthen responses to substantive information while reducing reliance on peripheral cues (Cao et al., 2021; K. Lee et al., 2025). In this study, involvement is treated as a cue-reweighting condition. It is not expected to increase responsiveness to every message characteristic in the same way. Rather, higher involvement should make substantive information more diagnostic and immediately accessible linguistic cues relatively less important.

Information richness provides users with factual and contextual material for evaluating a post. When involvement is high, users have stronger reasons to examine whether the information is sufficiently complete and useful. Richness should therefore become more strongly associated with dissemination behaviour as involvement increases.

**H5a:**  *User involvement strengthens the positive association between information richness and information dissemination behaviour.*

A similar pattern is expected for information relevance. Highly involved users are more motivated to determine whether a post addresses the event, their concerns, or its likely consequences. Relevant content should therefore carry greater weight in their dissemination decisions.

**H5b:**  *User involvement strengthens the positive association between information relevance and information dissemination behaviour.*

The same increase in elaboration motivation may reduce the relative importance of peripheral linguistic cues. When involvement is low, users may rely more heavily on features that make a message vivid, concrete, or conversational. These cues can support a quick judgment when users have limited motivation to evaluate the underlying content.

As involvement increases, however, users are more likely to focus on whether the information itself is useful, accurate, and applicable. Linguistic presentation may still help users understand a post, but it should become less decisive in the dissemination decision. This expectation is consistent with prior findings that involvement can weaken the relationship between peripheral cues and behavioural responses (H. Fu et al., 2021; Cao et al., 2021).

Specificity language may therefore matter less when users are highly involved, because concrete wording cannot substitute for substantive evaluation.

**H5c:**  *User involvement weakens the positive association between specificity language and information dissemination behaviour.*

Interactive language may also be more influential when users rely on readily accessible conversational cues. Highly involved users, by contrast, are more likely to base their dissemination decisions on the informational value of the post.

**H5d:**  *User involvement weakens the positive association between interactive language and information dissemination behaviour.*

## 4. Research Design and Empirical Analysis

### 4.1. Data Sources and Sample

This study uses the 2025 Tibet earthquake as its empirical setting. The event generated extensive discussion on Weibo and provides a clearly bounded context in which information production and public engagement can be observed over time. Such event-based cases are useful for examining communication processes under conditions of urgency and uncertainty (Zhou et al., 2016). Publicly available Weibo posts were collected using Gooseeker (V15.3.0) and the search term “Tibet earthquake”. The collection period extended from the onset of the event to 5 February 2025, covering the initial surge in attention and the subsequent decline in discussion. The initial dataset contained 68,166 posts.

The data were cleaned in three stages. First, duplicate records were removed. Second, posts unrelated to the focal earthquake and records containing only hyperlinks or hashtags were excluded. Third, three graduate students in information resource management reviewed records flagged as anomalous or potentially irrelevant. This manual review was conducted solely for data cleaning and relevance screening; it was not used to code the focal variables. The final analytical sample consisted of 51,856 posts. Table 1 presents four anonymized examples from the final dataset. The examples illustrate variation in message length, factual detail, interactive expression, and emergency guidance. Usernames, user IDs, and post URLs were removed to protect user privacy.

**Figure 2:**
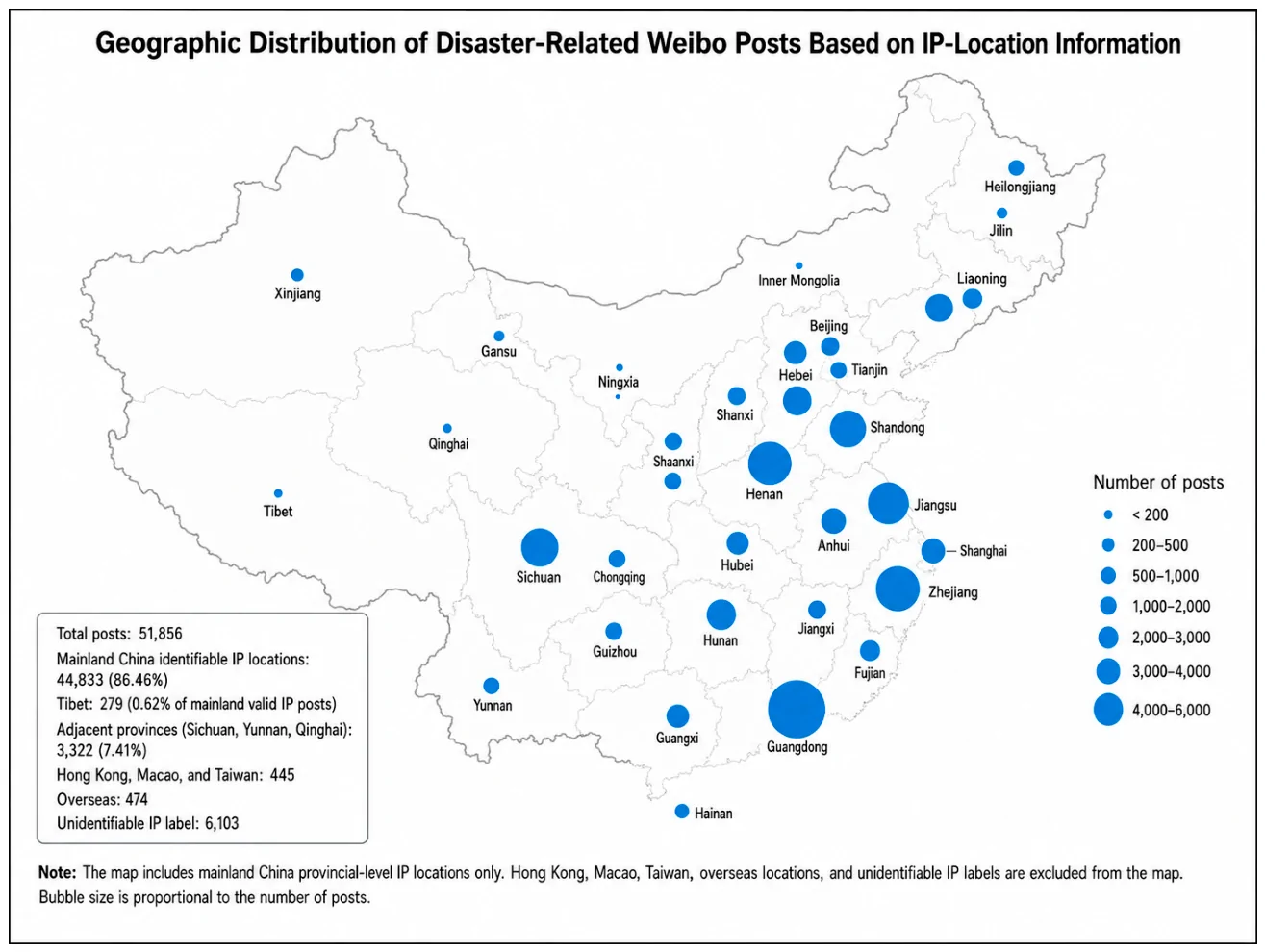
Geographic distribution of disaster-related Weibo posts based on IP-location information.

Province-level mainland IP-location information was available for 44,833 posts, accounting for 86.46% of the final sample. As shown in Figure 2, discussion extended well beyond the earthquake-affected area. Guangdong contributed the largest number of posts (*n* = 5644), followed by Henan (*n* = 3434), Shandong (*n* = 3014), Jiangsu (*n* = 2807), and Sichuan (*n* = 2474). Tibet accounted for 279 posts. Tibet, Sichuan, Yunnan, and Qinghai together contributed 3601 posts, representing 8.03% of posts with identifiable mainland IP locations. A further 445 posts were associated with Hong Kong, Macao, or Taiwan, and 474 were associated with overseas locations. Records with unidentifiable or other location labels were excluded from the province-level visualization. The geographic distribution indicates that the discussion reflected nationwide attention rather than activity confined to Tibet and neighbouring provinces.

### 4.2. Variable Measurement

The study uses naturally occurring social media data rather than survey or experimental measures. The focal variables are therefore operationalized through observable textual and structural characteristics of individual posts. These indicators capture expressed message characteristics and involvement, but they should not be interpreted as complete measures of underlying psychological constructs. TextMind, a Chinese-language text-analysis tool based on the Linguistic Inquiry and Word Count framework, was used to identify relevant linguistic categories (Lu et al., 2023; Ashokkumar & Pennebaker, 2022). Dictionary-based text analysis has been widely used to identify recurring linguistic patterns associated with psychological and communicative processes (Kim et al., 2024). TextMind reports normalized category frequencies, which were combined according to predefined measurement rules. All focal textual variables were generated automatically. Information richness was calculated from post length, URLs, and hashtags. Information relevance, specificity language, interactive language, and user involvement were calculated from predefined linguistic-category scores.

#### 4.2.1. Dependent Variable: Information Dissemination Behaviour

Information dissemination behaviour was measured using the total number of reposts and comments received by each post. Reposting extends the visibility of a message, whereas commenting reflects participation and discussion. Following Lu (2024), the two indicators were combined to represent overall dissemination. However, the raw count was highly right-skewed. Across the 51,856 posts, the mean was 4.950, the variance was 30,069.379, and the median was zero. A total of 82.09% of posts received neither reposts nor comments, whereas the maximum count was 33,906. The skewness coefficient was 152.519, indicating an exceptionally long right tail. The primary OLS analysis therefore used the natural logarithm of one plus the combined count:

*Beh_i_* = *ln*(1 + *Repostsi_i_* + *Comments_i_*)

This transformation retains zero-engagement posts and reduces the leverage of extremely high-engagement observations. The transformed variable represents relative dissemination intensity rather than the original number of reposts and comments.

#### 4.2.2. Independent Variables: Information Content and Language Style

(1) Information content

Information richness refers to the amount and structural diversity of information contained in a post. Longer posts provide more space for factual and contextual detail, while URLs extend access to external information and hashtags organize posts around identifiable topics. Drawing on M. Zhang et al. (2023) and Ding et al. (2014), information richness was calculated as

*Ric_i_* = *WordCount_i_* + *URL_i_* + *Hashtag_i_*

This indicator captures the observable amount and structural diversity of information in a post. It does not directly assess accuracy, credibility, or argument quality.

Information relevance refers to the extent to which a post contains contextual expressions connected to the focal disaster. Following C. H. Lee and Yu (2020), the measure combines normalized frequencies of spatial and future-oriented expressions:

*Rel_i_* = *Space_i_* + *Future_i_*

Spatial expressions locate events or actions, while future-oriented terms refer to anticipated developments, subsequent actions, or forthcoming conditions. The measure captures contextually expressed relevance rather than recipients’ subjective perceptions of relevance.

(2) Language Style

Specificity language refers to linguistic forms that provide concrete and contextual detail. Following Xie et al. (2024), the measure combines normalized frequencies of quantifiers, numerical expressions, and prepositions:

*Spe_i_* = *Quantifier_i_* + *Number_i_* + *Preposition_i_*

Quantifiers and numerical expressions specify amount, scale, or magnitude, while prepositions often locate entities and events within temporal, spatial, or relational contexts. The indicator captures linguistic specificity but does not directly measure perceived psychological distance.

Interactive language refers to expressions that create a sense of interpersonal exchange or invite participation. Following Abitbol and Lee (2017), the measure combines first-person plural pronouns, third-person plural pronouns, and interrogative markers:

*Int_i_* = *We_i_* + *They_i_* + *QuestionMark_i_*

Collective pronouns may frame a message in group-oriented terms, while interrogative markers can invite attention or response. The measure captures an interactive orientation in the post rather than actual reciprocal interaction between users.

#### 4.2.3. Moderating Variable: Involvement

User involvement refers to the extent to which a post expresses the author’s personal orientation toward the focal event. First-person singular pronouns have been used as linguistic indicators of self-focus and personal engagement with external events (Song & Zhu, 2025). User involvement was therefore measured using the normalized frequency of first-person singular pronouns. This indicator captures linguistically expressed involvement at the post level. It does not fully represent the author’s internal involvement, perceived risk, or personal experience of the disaster.

#### 4.2.4. Control Variables

Following Jin et al. (2024), the analysis controls for user, account, temporal, and geographic characteristics that may be associated with online engagement. Gender was coded as 1 for male and 0 for female. Certification type was coded as 2 for official accounts, 1 for individually verified accounts, and 0 for unverified accounts. Number of followers was measured using the account’s follower count. Activity level was measured using the user’s posting and interaction activity during the preceding day. IP location was coded as 1 when the reported location was Tibet, Qinghai, Yunnan, or Sichuan and 0 otherwise. Posting date records the calendar day on which the post was published, while posting time records the hour of publication on a 24 h scale. Variable definitions and measurements are summarized in Table 2.

### 4.3. Model Specification

The primary analysis uses ordinary least squares regression with heteroskedasticity-robust standard errors. The dependent variable is Beh, defined as the natural logarithm of one plus the total number of reposts and comments. Model (1) estimates the associations of the four focal message characteristics with dissemination intensity while controlling for user, account, temporal, and geographic characteristics. Model (2) adds user involvement and its interactions with the four message characteristics. Model (1) is used to test the main associations proposed in H1–H4, whereas Model (2) is used to examine the moderating role of user involvement proposed in H5a–H5d.

(1) $$M1:\;Beh=\alpha_{0}+\alpha_{1}Ric+\alpha_{2}Rel+\alpha_{3}Spe+\alpha_{4}Int+\alpha_{5}Control+\sigma_{1}$$

(2) $$\begin{array}{cc} M2:Beh=\beta_{0}+ & \beta_{1}Ric+\beta_{2}Rel+\beta_{3}Spe+\beta_{4}Int+\beta_{5}Inv+\beta_{6}Ric\cdot Inv+\beta_{7}Rel\\ & \cdot Inv+\beta_{8}Spe\cdot Inv+\beta_{9}Int\cdot Inv+\beta_{10}Control+\sigma_{2} \end{array}$$

Among these, Control represents the set of control variables; $\alpha_{0}$ and $\beta_{0}$ denote constant terms, while $\alpha_{1}∼\alpha_{4}$, $\beta_{1}∼\beta_{4}$ represent the estimated coefficients for the explanatory variables; $\beta_{5}$ denotes the estimated coefficient of the moderating variable; $\beta_{6}∼\beta_{9}$ represents the estimated coefficient for the interaction term between each explanatory variable and the moderating variable; $\alpha_{5}$ and $\beta_{10}$ are the estimated coefficients associated with the control variables; $\sigma_{1}∼\sigma_{2}$ stands for the residual term. The interaction terms test whether the associations of central-route and peripheral linguistic cues with dissemination intensity vary across levels of expressed user involvement. A positive interaction coefficient indicates that the association becomes stronger as involvement increases, whereas a negative coefficient indicates that the association becomes weaker.

OLS is used for the transformed measure of relative dissemination intensity. This specification facilitates the estimation and interpretation of multiple interaction terms while reducing the influence of extreme observations. Because the original combined number of reposts and comments is a non-negative count outcome characterized by substantial overdispersion, supplementary negative binomial regressions were estimated using the untransformed combined count as the dependent variable. The two estimation strategies therefore examined different specifications of the same dissemination outcome. OLS estimated associations with log-transformed dissemination intensity, whereas negative binomial regression estimated associations with the expected original dissemination count.

### 4.4. Descriptive Statistics and Correlations

The original dissemination count was highly right-skewed. Its mean was 4.950, whereas its variance was 30,069.379. The median was zero, 82.09% of the posts received neither reposts nor comments, and the maximum was 33,906. These distributional characteristics support the use of the logarithmically transformed outcome in the primary OLS analysis and the original count in the supplementary negative binomial analysis.

Table 3 reports the descriptive statistics and zero-order correlations for the variables used in the OLS models. The mean of the log-transformed dissemination measure was 0.302, with a standard deviation of 0.816. Information richness and interactive language were positively correlated with dissemination intensity (r = 0.117 and r = 0.060, respectively), whereas specificity language and user involvement were negatively correlated with it (r = −0.102 and r = −0.087, respectively). Information relevance had a small positive correlation with dissemination intensity (r = 0.047). The correlations among the focal predictors ranged from −0.171 to 0.543. The largest correlation was between information richness and user involvement (r = 0.543). Although this relationship is moderately strong, the correlation matrix does not by itself indicate severe multicollinearity. Variance inflation factors are therefore also examined in the regression analysis.

Given the large sample size, even small correlations may be statistically significant. The bivariate coefficients are consequently used to describe the direction and magnitude of the relationships rather than to determine substantive importance or hypothesis support. The hypotheses are evaluated using the multivariate regression estimates and supplementary model results.

### 4.5. Empirical Results

#### 4.5.1. Linguistic and Lexical Patterns of the Corpus

**Figure 3:**
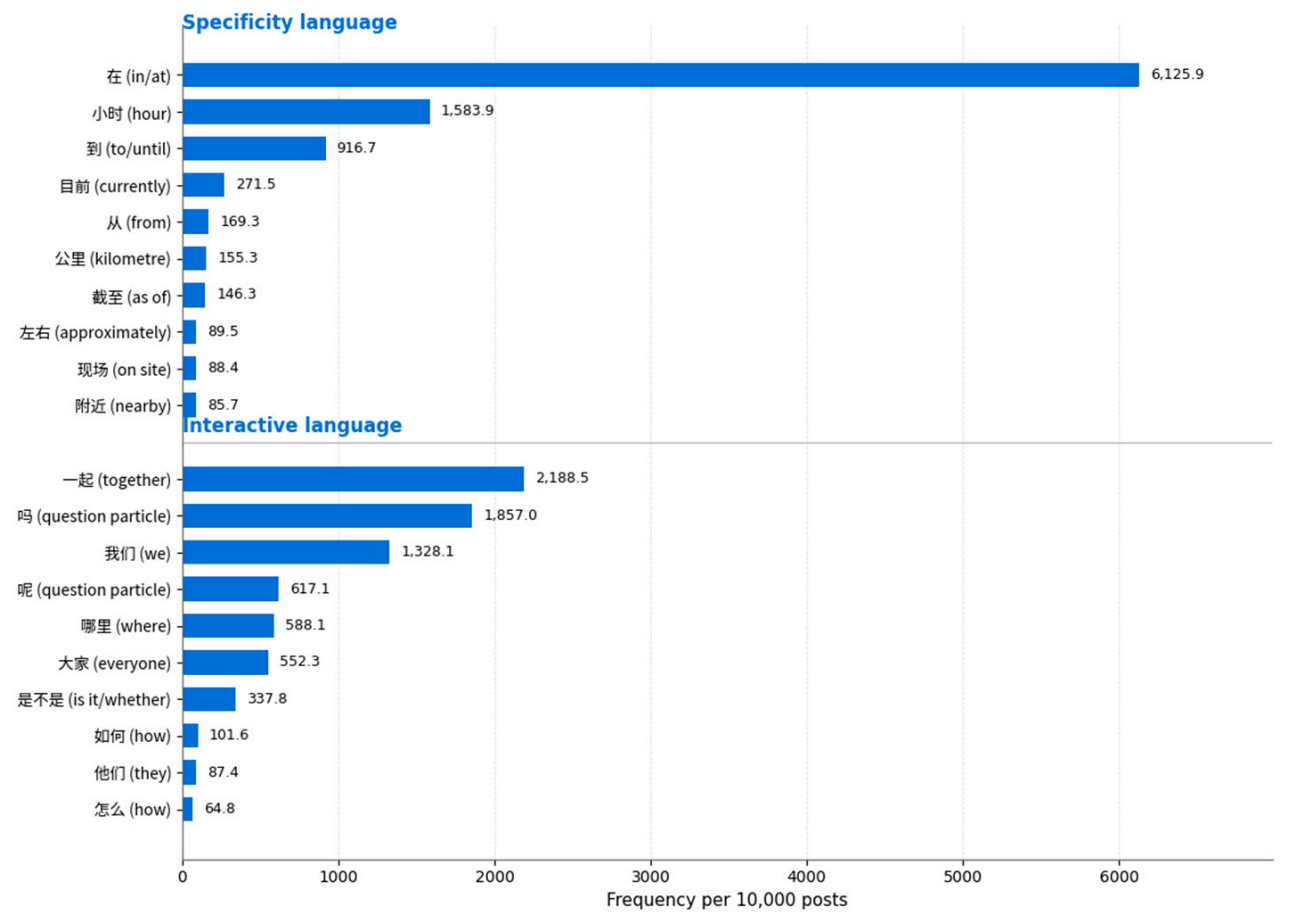
Word-level frequency patterns of specificity and interactive language.

Before estimating the regression models, we examined several descriptive features of the corpus to provide a clearer picture of how disaster-related information was expressed on Weibo. These analyses are intended to improve the transparency of the text-based measures rather than to serve as independent tests of the hypotheses. Figure 3 reports the frequencies of the linguistic categories used to construct specificity and interactive language. Specificity-related expressions included temporal, spatial, quantitative, and relational markers, whereas interactive expressions included collective pronouns and interrogative markers. Frequencies were standardized as occurrences per 10,000 posts to make the categories easier to compare. Specificity-related expressions were common throughout the corpus, particularly those referring to locations, time points, quantities, and situational relationships. Interactive expressions occurred less frequently but were still clearly present. The two groups therefore represent distinct linguistic features of the disaster-related posts.

**Figure 4:**
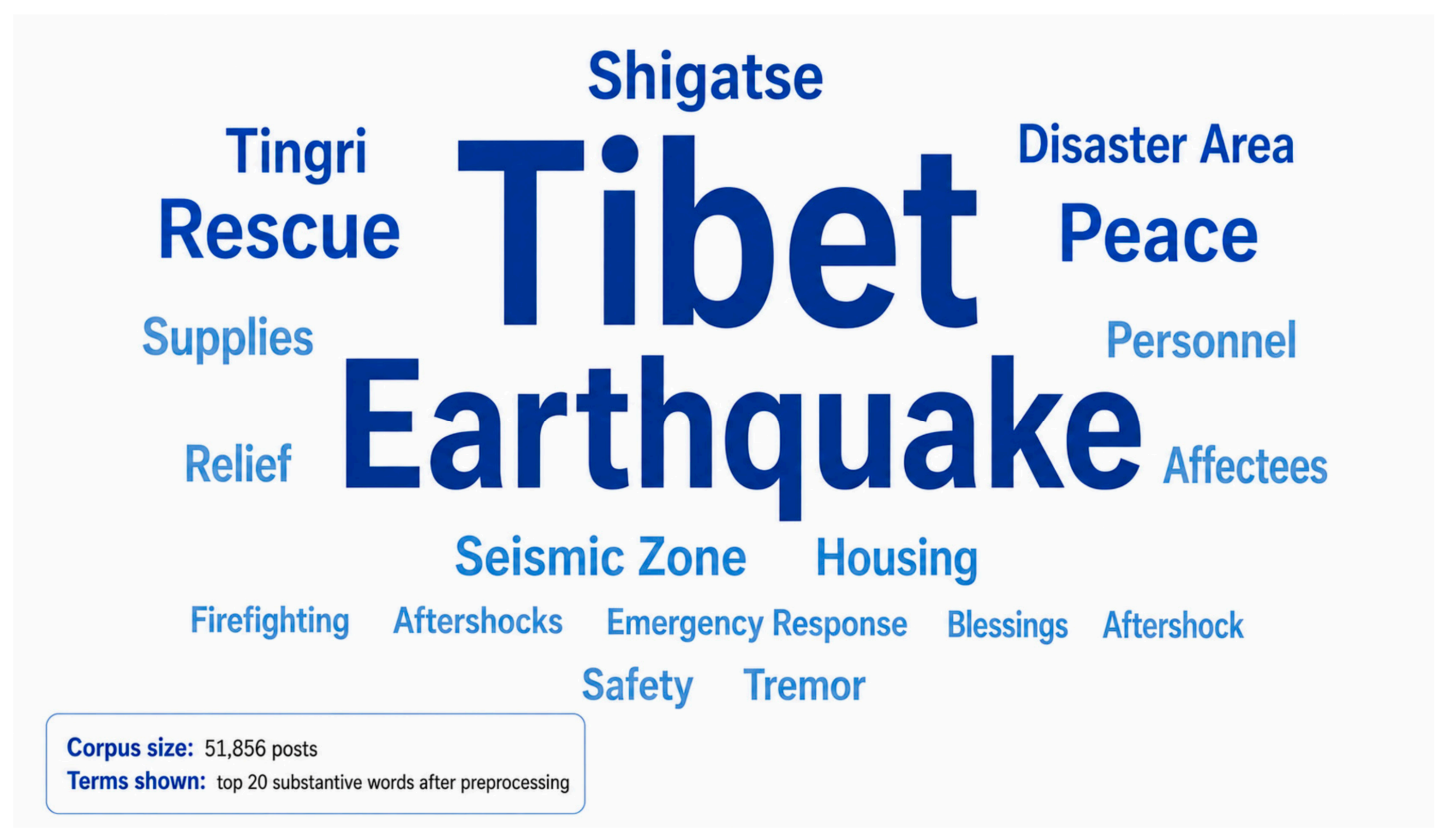
Word cloud of the most frequent substantive terms in the Weibo corpus.

A bag-of-words analysis was then conducted to summarize the broader lexical structure of the corpus. URLs, user mentions, platform-generated expressions, punctuation, non-substantive symbols, and common stop words were removed before Chinese word segmentation. Term frequencies were calculated across the full corpus, and the most frequent substantive terms were retained for visualization. As shown in Figure 4, the most prominent words concerned the earthquake, Tibet, safety, rescue, affected areas, and emergency response. This pattern indicates that the collected posts were closely centred on the focal event and its immediate public concerns. The word cloud is descriptive, however, and should not be interpreted as evidence that the theoretical constructs were directly observed or validated.

Table 4 summarizes affective and cognitive-process language identified by TextMind. Affective expressions accounted for 4.90% of the corpus, with positive-emotion language occurring more frequently than negative-emotion language. Cognitive-process expressions accounted for 8.92%, including language associated with certainty, insight, causation, and tentativeness. These percentages describe the linguistic composition of the corpus and do not directly measure users’ internal emotional or cognitive states.

#### 4.5.2. Baseline Regression Results

Table 5 reports the primary OLS estimates using log-transformed dissemination intensity as the dependent variable. Model (1) includes the four focal message characteristics and the control variables. Model (2) adds user involvement and the four interaction terms between user involvement and the message characteristics.

In Model (1), information richness was positively associated with overall dissemination intensity (β = 0.208, *p* < 0.001). Information relevance was also positively associated with dissemination intensity (β = 1.374, *p* < 0.001). These findings support H1 and H2. Specificity language showed a positive association with dissemination intensity (β = 2.285, *p* < 0.001), and interactive language was also positively associated with dissemination intensity (β = 6.541, *p* < 0.001). H3 and H4 were therefore supported in the primary OLS model.

The four coefficients should not be compared directly as measures of relative substantive importance because the predictors are operationalized on different scales. In particular, the larger raw coefficient for interactive language does not by itself establish that language style is universally more important than information content. The comparison is therefore interpreted cautiously and considered together with the supplementary model results.

#### 4.5.3. Moderating Effect Analysis

Model (2) examines whether the associations of message content and language style with dissemination intensity vary across levels of user involvement. The interaction between information richness and involvement was positive and statistically significant (β = 0.258, *p* < 0.001). This result indicates that the positive association between information richness and dissemination intensity became stronger as expressed involvement increased. H5a was therefore supported. The interaction between information relevance and involvement was also positive and significant (β = 1.282, *p* < 0.001). The positive association between information relevance and dissemination intensity was stronger at higher levels of involvement, supporting H5b. By contrast, the interaction between specificity language and involvement was negative and significant (β = −0.651, *p* < 0.01). The positive association between specificity language and dissemination intensity therefore weakened as involvement increased. H5c was supported. The interaction between interactive language and involvement was also negative and statistically significant (β = −11.585, *p* < 0.001). This finding indicates that interactive language was more strongly associated with dissemination intensity at lower levels of involvement and that the association became weaker as involvement increased. H5d was therefore supported.

**Figure 5:**
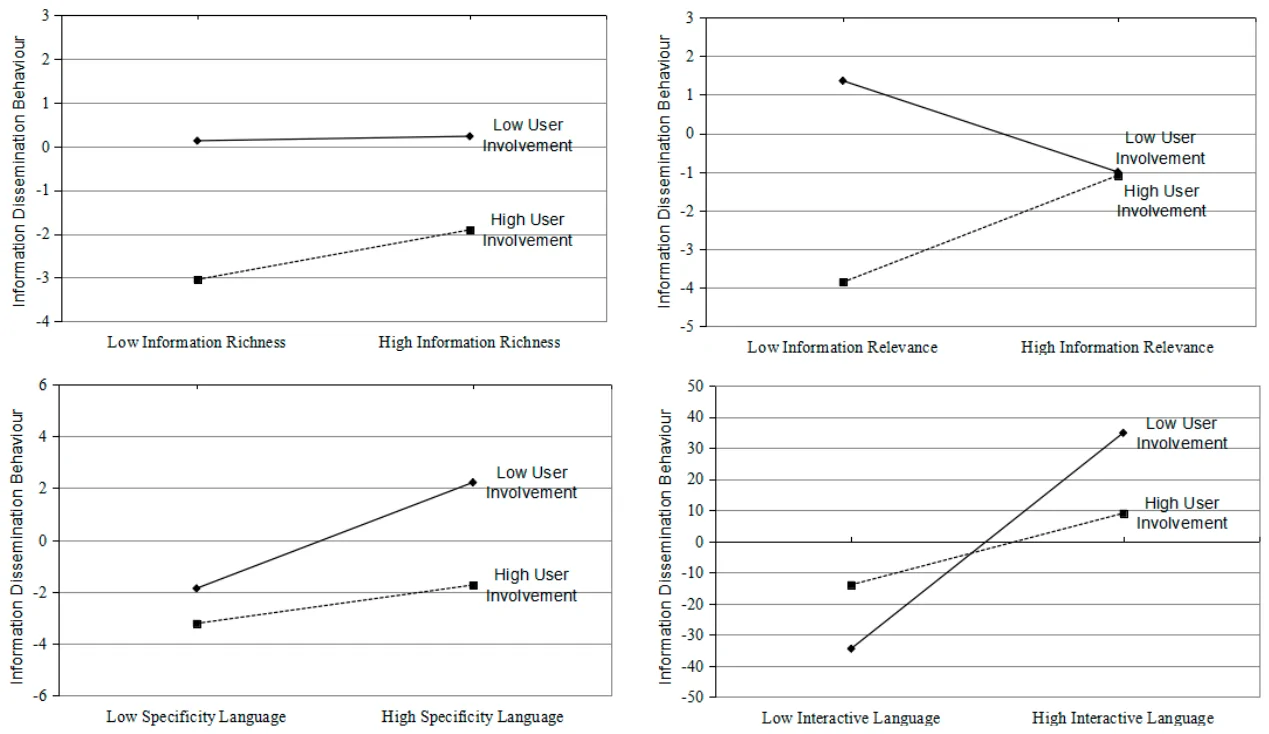
Moderation effect of user involvement.

Figure 5 presents the moderating effects of user involvement on the four focal associations. The slopes for information richness and information relevance become steeper as involvement increases, whereas the slopes for specificity and interactive language become flatter. These patterns are consistent with the proposed cue-reweighting account: expressed involvement is associated with greater relative weight being assigned to content-related cues and lower relative weight being assigned to readily accessible linguistic cues. The interaction plots should be interpreted as conditional associations rather than direct evidence that users consciously switched between processing routes. The study does not directly observe users’ internal elaboration processes.

#### 4.5.4. Robustness Analysis

(1) Winsorization and alternative transformation

To reduce the influence of extreme observations, the dependent variable was winsorized at the 1st and 99th percentiles. As shown in Table 6, information richness (β = 0.201, *p* < 0.001), information relevance (β = 1.311, *p* < 0.001), specificity language (β = 2.228, *p* < 0.001), and interactive language (β = 6.178, *p* < 0.001) remained positively associated with dissemination intensity. In the moderation model, the richness-involvement and relevance-involvement interactions remained positive, whereas the specificity-involvement and interactivity-involvement interactions remained negative. These estimates were consistent with the directions observed in the primary OLS models.

The robustness of the OLS findings was also assessed using a Box–Cox transformation of the dependent variable. Information richness, information relevance, specificity language, and interactive language remained positively associated with the transformed outcome in the main-effects model. The four interaction terms also retained the directions observed in the primary analysis. The winsorized and Box–Cox specifications therefore produced a pattern broadly consistent with the baseline OLS results.

(2) Negative binomial models

Because the original combined number of reposts and comments is a non-negative count variable characterized by substantial overdispersion, a supplementary negative binomial regression was estimated. The dependent variable was the untransformed total number of reposts and comments received by each post. The model used the same sample, predictors, interaction terms, and control variables as the primary OLS analysis. Table 7 reports the full negative binomial regression results.

The estimated dispersion parameters were 16.940 in the main-effects model and 16.258 in the moderation model. These values indicate substantial overdispersion and support the use of negative binomial rather than Poisson regression.

In the main-effects model, information richness was positively associated with the overall dissemination count (β = 1.550, SE = 0.175, *p* < 0.001). Interactive language was also positively associated with dissemination (β = 48.146, SE = 5.988, *p* < 0.001). Information relevance was not statistically significant (β = −0.817, SE = 1.673, *p* = 0.626), whereas specificity language was negatively associated with dissemination count (β = −13.237, SE = 2.001, *p* < 0.001).

In the moderation model, information richness remained positively associated with dissemination count (β = 1.199, SE = 0.193, *p* < 0.001), and interactive language remained positively associated with dissemination (β = 123.983, SE = 18.548, *p* < 0.001). Information relevance (β = −9.175, SE = 2.533, *p* < 0.001) and specificity language (β = −8.479, SE = 2.823, *p* = 0.003) were negatively associated with dissemination count when involvement was at its reference level. The richness-involvement interaction was positive and significant (β = 1.616, SE = 0.231, *p* < 0.001), as was the relevance-involvement interaction (β = 12.235, SE = 2.753, *p* < 0.001). The specificity-involvement interaction was negative but only marginally significant (β = −4.659, SE = 2.792, *p* = 0.095). The interactivity-involvement interaction was negative and statistically significant (β = −50.480, SE = 13.010, *p* < 0.001).

Overall, the negative binomial results partially support the primary OLS findings. The positive associations of information richness and interactive language, together with the positive richness- and relevance-involvement interactions and the negative interactivity-involvement interaction, were stable across specifications. By contrast, the estimates for information relevance and specificity language differed between the logarithmically transformed OLS model and the original-count negative binomial model. The specificity-involvement interaction retained the predicted negative direction but was only marginally significant.

## 5. Discussion and Conclusions

### 5.1. Discussion of the Main Findings

This study examined how information content and language style are associated with disaster-information dissemination on Weibo and whether these associations vary with user involvement. The findings show that message content and linguistic presentation both matter, but their relationships with dissemination are not equally stable across model specifications.

Regarding RQ1, the primary OLS results indicate that information richness, information relevance, specificity language, and interactive language are all positively associated with log-transformed dissemination intensity. These findings are consistent with previous research showing that both informational value and linguistic presentation are related to public engagement during crises (L. Zhang et al., 2014; C. H. Lee & Yu, 2020). Richer posts may give users more factual and contextual material on which to assess message utility, while interactive expressions may make a post appear more conversational and response-oriented (Abitbol & Lee, 2017; Y. Wang et al., 2024). The negative binomial results, however, provide a more qualified picture. Information richness and interactive language remain positively associated with the original combined count of reposts and comments, whereas information relevance is not positively associated with the count outcome and specificity language shows a negative association. The divergence suggests that some message characteristics may be associated differently with relative dissemination intensity and with the original volume of engagement. It also cautions against treating findings from a single outcome specification as universally stable.

Regarding RQ2, information richness and interactive language show the most consistent positive associations across the OLS and negative binomial models. The stability of information richness reinforces the importance of providing sufficiently detailed and contextually useful information during rapidly evolving disasters (L. Zhang et al., 2014; Y. Wang et al., 2024). The consistent association of interactive language also supports earlier evidence that conversational and participatory expressions can encourage online engagement (Abitbol & Lee, 2017; C. H. Lee & Yu, 2020). By contrast, the results for information relevance and specificity language are more sensitive to the treatment of the dependent variable. One possible explanation is that the relevance measure captures spatial and future-oriented expressions rather than recipients’ subjective judgments of relevance. Similarly, specificity may improve the clarity of a post without necessarily being associated with a larger number of reposts and comments. The findings therefore do not support a broad claim that language style is more important than message content. Because the focal variables were measured on different scales, their raw coefficients cannot be compared directly as indicators of substantive importance.

Regarding RQ3, user involvement changes the relative importance of content-related and linguistic cues. In the OLS model, involvement strengthens the positive associations of information richness and relevance while weakening those of specificity and interactive language. The negative binomial model reproduces the positive richness-involvement and relevance-involvement interactions and the negative interactivity-involvement interaction. The specificity-involvement interaction retains the expected negative direction but is only marginally significant. This pattern is broadly consistent with ELM. When users express greater involvement, they may be more motivated to evaluate whether a post contains useful and situationally relevant information, making content-related cues more diagnostic (Petty & Cacioppo, 1986). At the same time, readily accessible linguistic cues may become relatively less important. Similar patterns have been reported in previous studies showing that involvement can strengthen responses to substantive information while reducing reliance on peripheral characteristics (Cao et al., 2021; H. Fu et al., 2021).

The findings therefore provide qualified support for the proposed cue-reweighting mechanism. User involvement does not appear to increase responsiveness to every message characteristic in the same way. Instead, it changes which cues are more closely associated with dissemination behaviour. This interpretation concerns observable linguistic and behavioural patterns rather than users’ internal processing states, which were not directly measured. CLT helps clarify the representational properties of the linguistic cues in this framework. Specificity language provides concrete and contextual detail, whereas interactive language creates interpersonal orientation and communicative immediacy (Trope & Liberman, 2010).

### 5.2. Theoretical Contributions

This study makes three theoretical contributions. First, it brings message content and linguistic presentation into the same framework for explaining disaster-information dissemination. Previous research has often examined information quality, message content, emotional expression, or language style separately (L. Zhang et al., 2014; C. H. Lee & Yu, 2020). By analysing information richness, information relevance, specificity language, and interactive language together, this study shows that dissemination is associated with both what a message communicates and how that information is expressed. This integrated perspective offers a more complete account of communication behaviour during sudden disasters.

Second, the study develops a cue-reweighting account of user involvement. ELM suggests that involvement increases motivation to scrutinize substantive information and reduces reliance on accessible peripheral cues (Petty & Cacioppo, 1986). The present findings refine this argument by showing that involvement is associated with changes in the relative diagnostic value of different message characteristics. Higher involvement strengthens the associations of content-related cues and weakens the association of interactive language. Involvement therefore appears to change which cues matter more rather than simply increasing overall responsiveness.

Third, the study clarifies the respective roles of ELM and CLT. ELM provides the processing architecture for distinguishing central-route and peripheral cues, whereas CLT helps characterize the concrete, contextual, and interpersonal properties of linguistic presentation (Trope & Liberman, 2010). This distinction avoids treating CLT as an additional processing route or assuming that specificity directly changes psychological distance. The study also extends ELM from attitudes and behavioural intentions to observable dissemination behaviour in social media settings.

### 5.3. Practical Implications

The findings offer three practical implications for disaster communication. First, emergency communicators should prioritize substantive and sufficiently rich information. Information richness is positively associated with dissemination in both the OLS and negative binomial models. Disaster-related posts should therefore provide factual updates, contextual explanations, resource information, and actionable guidance rather than relying mainly on brief symbolic statements. This recommendation is consistent with previous research showing that useful and informative crisis messages are more likely to attract public attention and sharing (L. Zhang et al., 2014; Y. Wang et al., 2024). For enterprises, communication directed at highly involved stakeholders should emphasize accurate, complete, and internally consistent information, as gaps or inconsistencies may undermine trust.

Second, message design should take audience involvement into account. Users expressing greater involvement appear more responsive to information richness and relevance. Communication directed at affected residents, family members, volunteers, or users closely following the event should therefore emphasize completeness, situational usefulness, and practical value. For enterprises, when involvement is low, interactive and specific linguistic cues can serve as low-cost tools to lower processing difficulty and activate cognitive engagement. For less involved stakeholders, specific and interactive language may help attract attention and improve message accessibility, but it should complement rather than replace substantive information.

Third, linguistic specificity should be used purposefully rather than mechanically. Temporal, spatial, and quantitative details can improve clarity and support verification or action, but the negative binomial results show that specificity is not uniformly associated with higher original engagement counts. Communicators should therefore include concrete details when they improve comprehension or decision-making, not simply to increase visibility. Communication performance should also be assessed through more than one engagement indicator because transformed dissemination intensity and original engagement counts capture different aspects of public response. Enterprises may also adjust the balance between content and language as stakeholder attention changes, using real-time engagement indicators to inform, rather than determine, communication decisions.

### 5.4. Research Limitations and Future Research Directions

Several limitations should be considered when interpreting the findings. The observational design identifies conditional associations rather than causal effects, and unobserved factors such as source credibility, media format, account history, prior exposure, and platform recommendation mechanisms may also shape dissemination behaviour. The focal variables are also computational proxies: spatial and future-oriented expressions do not fully represent perceived relevance, and first-person singular pronouns capture only one linguistic aspect of involvement. CLT was used to characterize the representational properties of specificity and interactive language, as psychological distance was not directly measured. Finally, the study focuses on one disaster and one social media platform, and several estimates vary across model specifications. Future research could combine text analysis with surveys, experiments, or quasi-experimental designs, directly measure psychological distance and involvement, and compare different events, platforms, and forms of engagement to test the generalizability and boundary conditions of the proposed framework.

## Figures and Tables

**Table 1:** Examples of disaster-related Weibo posts in the dataset.

Example	Original Chinese Post	English Translation
1	#西藏地震#平平安安	#TibetEarthquake# May everyone stay safe.
2	#西藏地震#藏区的宝宝们，平平安安，注意安全。	#TibetEarthquake# To everyone in the affected Tibetan areas, please stay safe and take care.
3	#西藏地震#1月7日9时5分在西藏日喀则市定日县（北纬28.54度，东经87.43度）发生6.8级左右地震。	#TibetEarthquake# At 9:05 a.m. on January 7, an earthquake of approximately magnitude 6.8 occurred in Dingri County, Shigatse, Tibet, at 28.54° N and 87.43° E.
4	【#地震来临这样保护自己#】地震来袭时应迅速前往室外开阔处；若无法及时撤离，应躲到坚实家具下或承重墙角，并保护头部。你学会了吗？	#How to Protect Yourself during an Earthquake# Move quickly to an open outdoor area when possible. If evacuation is not possible, shelter under sturdy furniture or beside a load-bearing wall and protect your head. Have you learned these safety measures?

**Table 2:** Variables design.

Variable			Symbol	Measurement
Dependent Variable		Information Dissemination Behaviour	Beh	Natural logarithm of one plus the total number of reposts and comments.
Independent Variables	Information Content	Information Richness	Ric	Word count plus the numbers of URLs and hashtags.
		Information Relevance	Rel	Sum of normalized spatial and future-oriented language scores.
	Language Style	Specificity Language	Spe	Sum of normalized quantifier, number, and preposition scores.
		Interactive Language	Int	Sum of normalized first-person plural, third-person plural, and interrogative-marker scores.
Moderating Variable		User Involvement	Inv	Normalized frequency of first-person singular pronouns.
Control Variables		User Gender	Sex	The gender of Weibo users is coded as 1 for male and 0 for female.
		Certification Type	Cer	Certification type was coded as 2 for official accounts, 1 for individually verified accounts, and 0 for unverified accounts.
		Number of Followers	Att	The total number of followers of the user.
		Activity Level	Liv	The user’s total number of posts published and interactions performed on the previous day.
		IP Location	Dis	Weibo user’s IP location: assigned a value of 1 if located in Tibet, Qinghai, Yunnan, or Sichuan, and 0 otherwise.
		Post Date	Day	The calendar day the post was published (1 to 31).
		Post Time	Tim	The hour the post was published, on a 24 h scale (0 to 23).

**Table 3:** Descriptive statistics and correlation analysis.

Variable	Mean	SD	Min	Max	1	2	3	4	5	6	7	8	9	10	11	12	13
1. Log-transformed dissemination intensity	0.302	0.816	0.000	10.431	1												
2. Information richness	4.237	0.415	2.398	5.004	0.117 *	1											
3. Information relevance	0.045	0.030	0.000	0.255	0.047 *	0.064 *	1										
4. Specificity language	0.054	0.027	0.000	0.206	−0.102 *	0.244 *	0.356 *	1									
5. Interactive language	0.002	0.007	0.000	0.111	0.060 *	0.101 *	−0.153 *	−0.098 *	1								
6. User involvement	0.934	0.782	0.000	2.996	−0.087 *	0.543 *	−0.171 *	0.197 *	0.203 *	1							
7. Gender	0.245	0.430	0.000	1.000	0.225 *	0.014 *	0.121 *	−0.176 *	−0.022 *	−0.288 *	1						
8. Certification type	0.866	0.702	0.000	2.000	0.190 *	0.206 *	0.014 *	−0.304 *	0.038 *	−0.222 *	0.256 *	1					
9. Number of followers	6.175	1.139	0.000	9.904	0.140 *	0.232 *	−0.083 *	−0.060 *	0.091 *	0.159 *	0.077 *	0.187 *	1				
10. Activity level	11.066	5.690	0.000	23.016	0.024 *	0.060 *	−0.031 *	−0.034 *	0.019 *	0.007	−0.027 *	0.110 *	0.095 *	1			
11. IP location	0.069	0.254	0.000	1.000	−0.032 *	−0.039 *	−0.007	0.006	0.003	0.016 *	−0.049 *	−0.071 *	−0.037 *	0.011 *	1		
12. Posting date	11.765	5.177	1.000	31.000	−0.033 *	0.291 *	−0.137 *	0.051 *	0.155 *	0.347 *	−0.066 *	0.071 *	0.176 *	−0.019 *	−0.015 *	1	
13. Posting time	13.429	6.251	0.000	23.000	−0.006	−0.008	−0.041 *	−0.051 *	0.059 *	0.010 *	−0.019 *	0.026 *	0.002	0.011 *	0.009 *	−0.013 *	1

Note: * indicates statistical significance at *p* < 0.05.

**Table 4:** Descriptive statistics of affective and cognitive-process language.

TextMind Category	Mean Proportion
Affective processes	4.90%
Positive emotion	3.67%
Negative emotion	0.47%
Anxiety	0.10%
Anger	0.09%
Sadness	0.09%
Cognitive processes	8.92%
Certainty	1.02%
Insight	0.71%
Causation	0.67%
Tentativeness	0.46%

**Table 5:** Model estimation results.

Variable	Dependent Variable: Information Dissemination Behaviour	
	Model (1)	Model (2)
Information Richness	0.208 *** (22.14)	0.310 *** (25.14)
Information Relevance	1.374 *** (10.92)	0.101 (0.51)
Specificity Language	2.285 *** (14.82)	1.392 *** (6.55)
Interactive Language	6.541 *** (12.53)	23.075 *** (15.53)
User Involvement		−1.327 *** (−16.60)
Information Richness × User Involvement		0.258 *** (15.21)
Information Relevance × User Involvement		1.282 *** (6.07)
Specificity Language × User Involvement		−0.651 ** (−2.77)
Interactive Language × User Involvement		−11.585 *** (−10.13)
User Gender	0.323 *** (38.34)	0.280 *** (32.23)
Certification Type	0.100 *** (17.95)	0.022 ** (3.41)
Number Of Followers	0.067 *** (21.22)	0.066 *** (20.94)
Activity Level	0.000 (0.27)	0.000 (−0.08)
IP Location	−0.034 * (−2.54)	−0.038 ** (−2.81)
Post Date	−0.012 *** (−16.35)	−0.010 *** (−13.39)
Post Time	−0.001 * (−2.49)	−0.001 ** (−2.60)
Constant	−0.958 *** (−24.87)	−1.148 *** (−22.89)
R^2^	0.097	0.108

Note: Model (1) includes the four focal message characteristics and control variables. Model (2) additionally includes user involvement and the four interaction terms. Robust t-statistics are reported in parentheses. *, **, and *** indicate *p* < 0.05, *p* < 0.01, and *p* < 0.001, respectively.

**Table 6:** Robustness analyses using winsorized and Box–Cox-transformed dependent variables.

Variable	Winsorization		Box–Cox Transformation	
	M1	M2	M3	M4
Information Richness	0.201 *** (23.29)	0.288 *** (25.46)	0.336 *** (29.36)	0.354 *** (23.64)
Information Relevance	1.311 *** (11.34)	0.016 (0.09)	1.492 *** (9.77)	0.006 (0.02)
Specificity Language	2.228 *** (15.73)	1.365 *** (6.99)	3.458 *** (18.46)	1.657 *** (6.41)
Interactive Language	6.178 *** (12.88)	21.482 *** (15.74)	9.093 *** (14.34)	28.262 *** (15.66)
User Involvement		−1.285 *** (−17.50)		−2.14 *** (−22.04)
Information Richness × User Involvement		0.253 *** (16.20)		0.45 *** (21.82)
Information Relevance× User Involvement		1.099 *** (5.66)		1.411 *** (5.50)
Specificity Language× User Involvement		−0.606 ** (−2.81)		−1.39 *** (−4.88)
Interactive Language× User Involvement		−10.716 *** (−10.20)		−13.581 *** (−9.77)
User Gender	0.307 *** (39.70)	0.268 *** (33.66)	0.381 *** (37.31)	0.353 *** (33.50)
Certification Type	0.091 *** (17.76)	0.018 ** (2.95)	0.121 *** (17.85)	0.028 *** (3.59)
Number Of Followers	0.067 *** (23.11)	0.066 *** (22.69)	0.101 *** (26.22)	0.094 *** (24.52)
Activity Level	0.000 (0.16)	0.000 (−0.21)	0.003 ** (3.41)	0.002 ** (3.02)
IP Location	−0.033 ** (−2.65)	−0.036 ** (−2.96)	−0.035 * (−2.12)	−0.045 ** (−2.74)
Post Date	−0.011 *** (−16.46)	−0.009 *** (−13.77)	−0.011 *** (−13.33)	−0.012 *** (−13.25)
Post Time	−0.001 ** (−2.57)	−0.001 ** (−2.72)	−0.001 (−1.44)	−0.001 (−1.95)
Constant	−0.938 *** (−26.49)	−1.083 *** (−23.51)	−2.02 *** (−43.17)	−1.831 *** (−30.05)
R^2^	0.103	0.1148	0.113	0.124

Note, *** *p* < 0.001; ** *p* < 0.01; * *p* < 0.05.

**Table 7:** Robustness analyses using negative binomial regression.

Variables	M1	M2
Information richness	1.550 *** (0.175)	1.199 *** (0.193)
Information relevance	−0.817 (1.673)	−9.175 *** (2.533)
Specificity language	−13.237 *** (2.001)	−8.479 ** (2.823)
Interactive language	48.146 *** (5.988)	123.983 *** (18.548)
User involvement		−8.596 *** (1.095)
Richness × involvement		1.616 *** (0.231)
Relevance × involvement		12.235 *** (2.753)
Specificity × involvement		−4.659 ^†^ (2.792)
Interactivity × involvement		−50.480 *** (13.01)
Number of followers	0.027 (0.041)	0.03 (0.041)
Activity level	−0.008 (0.009)	−0.007 (0.009)
Gender	1.092 *** (0.122)	1.184 *** (0.125)
Certification type	1.137 *** (0.103)	0.983 *** (0.101)
IP location	0.224 (0.344)	−0.059 (0.252)
Posting date	−0.036 ** (0.012)	−0.032 * (0.014)
Posting time	−0.007 (0.007)	−0.008 (0.006)
Constant	−5.495 *** (0.765)	−3.662 *** (0.897)
Log likelihood	−50,859.200	−50,564.837
pseudo_r2	0.0539	0.0594
wald_chi2	855.55 ***	914.19 ***
alpha	16.94	16.258
AIC	101,746.40	101,165.67
BIC	101,870.39	101,325.09

Note, *** *p* < 0.001; ** *p* < 0.01; * *p* < 0.05, † *p* < 0.1.

## Data Availability

An anonymized sample of the dataset is publicly available in Zenodo at https://doi.org/10.5281/zenodo.21509363. The complete dataset is not publicly shared because it contains user-generated social media content and is subject to platform terms and privacy considerations.
